# The relationship between subjective sleep quality and cognitive performance in healthy young adults: Evidence from three empirical studies

**DOI:** 10.1038/s41598-020-61627-6

**Published:** 2020-03-17

**Authors:** Zsófia Zavecz, Tamás Nagy, Adrienn Galkó, Dezso Nemeth, Karolina Janacsek

**Affiliations:** 1 Doctoral School of Psychology, ELTE Eötvös Loránd University, Budapest, Hungary; 2 Institute of Psychology, ELTE Eötvös Loránd University, Budapest, Hungary; 3 Institute of Cognitive Neuroscience and Psychology, Hungarian Academy of Sciences, Budapest, Hungary; 4 Lyon Neuroscience Research Center (CRNL), INSERM, CNRS, Université Claude Bernard Lyon 1, Lyon, France; 5 School of Human Sciences, Faculty of Education, Health and Human Sciences, University of Greenwich, London, United Kingdom

**Keywords:** Sleep, Working memory, Human behaviour

## Abstract

The role of subjective sleep quality in cognitive performance has gained increasing attention in recent decades. In this paper, our aim was to test the relationship between subjective sleep quality and a wide range of cognitive functions in a healthy young adult sample combined across three studies. Sleep quality was assessed by the Pittsburgh Sleep Quality Index, the Athens Insomnia Scale, and a sleep diary to capture general subjective sleep quality, and the Groningen Sleep Quality Scale to capture prior night’s sleep quality. Within cognitive functions, we tested working memory, executive functions, and several sub-processes of procedural learning. To provide more reliable results, we included robust frequentist as well as Bayesian statistical analyses. Unequivocally across all analyses, we showed that there is no association between subjective sleep quality and cognitive performance in the domains of working memory, executive functions and procedural learning in healthy young adults. Our paper can contribute to a deeper understanding of subjective sleep quality and its measures, and we discuss various factors that may affect whether associations can be observed between subjective sleep quality and cognitive performance.

## Introduction

There is a widely accepted belief that experiencing poor sleep quality, including subjective experiences (e.g., reporting difficulties falling asleep, waking up frequently during the night, or feeling tired during the day), indisputably decreases cognitive performance. We can often hear people complaining about weaker memory and/or attentional performance in relation to their experienced sleep insufficiency. This phenomenon can be particularly prevalent amongst university students since the pressure for academic performance in this population is exceptionally high. The possible overestimation of the importance of one’s subjective sleep quality can even lead to placebo or nocebo effects on cognitive performance^1,2^. However, scientific evidence on the relationship between experienced subjective sleep quality and cognition is still inconclusive^3–7^. Therefore, our aim in the current study was to test whether subjective sleep quality is associated with cognitive performance in healthy young adults.

The role of sleep in cognitive performance has gained increasing attention in neuroscience and sleep research in recent decades^8,9^. Numerous experimental methods exist that can be employed for examining the association between sleep and cognitive performance. Sleep parameters can be evaluated based on actigraph or electroencephalograph measurements (i.e., objective measures), which are time-consuming and require expensive equipment. Hence, researchers and clinicians often tend to rely on questionnaires (i.e., subjective measures) to assess sleep parameters (e.g., sleep latency, sleep quality, sleep disturbances, or sleep duration). This inclination has also motivated the current study to explore the relationship between sleep questionnaires and cognitive functions.

Previous studies have shown that subjective and objective sleep parameters, such as sleep latency, sleep duration, or sleep efficiency could differ^10–12^; the strength of correlation between the subjective and objective measures of the same parameters varied between 0.21 and 0.62 for sleep latency and duration, while it was close to 0 for sleep efficiency. Subjective sleep quality can vary from objective sleep quality as it is typically estimated from a combination of parameters, such as sleep initiation, sleep continuity (number of awakenings), and/or depth of sleep. For instance, extreme deviations can occur between subjective and objective measures in sleep disorders, such as insomnia or sleep-state misperception. According to Zhang and Zhao^13^, the subjective and objective measures together should determine the type of treatment and medication in sleep disorders. Stepanski *et al*.^7^ showed that, within insomniac patients, the decisive factor of whether a patient seeks medication is their subjective evaluation of their sleep quality and daytime functioning. Furthermore, Gavriloff *et al*.^1^ found that providing sham feedback about their sleep to patients with insomnia influenced their daytime symptoms and performance in attention and vigilance tasks. Similarly, in a placebo sleep study, young adults were randomly told they had below or above average sleep quality based on their brainwaves and other psychophysiological measures^2^. This constructed belief about their sleep quality affected their performance in attentional and executive function tasks. Thus, beyond therapeutic importance, it appears that subjective sleep quality can have further explanatory value for cognitive performance compared to objective measures.

One of the most widely-used sleep questionnaires is the Pittsburgh Sleep Quality Index (PSQI)^14^, a self-administered questionnaire, in which participants rate their subjective sleep quality based on several questions. These questions deal with various aspects of sleep that range from the average amount of sleep during the night, the difficulty experienced in falling asleep, and other sleep disturbances. Nevertheless, there are other popular measurements, such as the Athens Insomnia Scale (AIS)^15^, which measures difficulties in falling asleep or maintaining sleep, as well as sleep diaries, which capture the sleeping habits of the participants from day to day, spanning a few days or weeks. Sleep questionnaires and sleep diaries are two different types of self-reported measures: while sleep questionnaires are administered at a single point in time, and ask about various aspects of sleep experience in a longer time period retrospectively, sleep diaries are ongoing, daily self-monitoring tools. Libman *et al*.^16^ showed that the two measurement types are tapping the same domains but lead to somewhat different results due to methodological differences: questionnaires can be susceptible to memory distortion while sleep diaries may be distorted by atypical sleep experiences during the monitored period.

Previous research on subjective sleep quality and cognitive performance has led to mixed findings. While some studies focusing on healthy participants have shown that poorer sleep quality as measured by the PSQI score was associated with weaker working memory^4^, executive functions^5^, and decision-making performance^17^, others have failed to find an association between subjective sleep quality and cognitive performance^6,7^. Bastien *et al*.^3^ showed different associations between subjective sleep quality as measured by a sleep diary and cognitive performance in patients with insomnia who received or did not receive treatment and in elderly participants who reported good sleep quality. Interestingly, in good sleepers, greater subjective depth, quality, and efficiency of sleep were associated with better performance on attention and concentration tasks but poorer memory performance. These findings suggest that further studies are needed to clarify the complex relationship between subjective sleep quality and aspects of cognitive functioning.

Notably, these previous studies focused on diverse populations, including adolescents, elderly and clinical groups, and relied on sample sizes ranging from around 20 to 100, with smaller sample sizes potentially limiting the robustness of the observed results. In these studies, subjective sleep quality was assessed by a combination of self-reported measures, such as difficulty in sleep initiation, sleep continuity, and/or depth of sleep. In contrast to subjective sleep quality captured by a combination of such measures, self-reported sleep duration has been studied more thoroughly. In a large study with more than 100,000 participants, Sternberg *et al*.^18^ reported a quadratic relationship between self-reported sleep duration and performance in cognitive tasks assessing working memory and arithmetics. Furthermore, a recent powerful meta-analysis focusing on elderly participants also showed that both short and long sleep increased the odds of poor cognitive performance^19^. A similar association was shown in another study investigating insomnia symptoms and cognitive performance in a large sample of participants^20^: self-reported sleep duration extremes were associated with impaired performance. Systematic investigations on the relationship between subjective sleep quality as captured by a combination of parameters (such as sleep latency, subjective sleep quality, sleep disturbances) and cognitive performance using larger sample sizes are, however, still lacking.

Moreover, in previous investigations focusing on the association between subjective sleep quality and various aspects of cognitive performance, the potential relationship with procedural learning/memory has largely been neglected. The procedural memory system underlies the learning, storage, and use of cognitive and perceptual-motor skills and habits^21^. Evidence suggests that the system is multifaceted in that it supports numerous functions that are performed automatically, including sequences, probabilistic categorization, and grammar, and perhaps aspects of social skills^22–26^. Considering the importance of this memory system, the clarification of its relationship with subjective sleep quality would be indispensable.

Here we aimed to fill the gaps identified in previous research by providing an extensive investigation on the relationship between subjective sleep quality and cognitive performance in healthy young adults. Within cognitive functions, we focused on working memory, executive functions, and procedural learning. We chose these domains because 1) the relationship between working memory, executive functions and subjective sleep quality has remained inconclusive, and 2) the relationship between procedural learning/memory and subjective sleep quality has largely been neglected in previous studies. Therefore, in the latter case, we explored several measures of procedural learning in order to obtain a more detailed picture of the potential associations with subjective sleep quality. To increase the robustness of our analyses, we created a database of 235 participants’ data by pooling three separate datasets from our lab. We assessed subjective sleep quality by PSQI and AIS (Study 1–3), Groningen Sleep Quality Scale (GSQS, Study 2), and a sleep diary (Study 2). These separate measures capture somewhat different aspects of self-reported sleep quality and thus provide a detailed picture. We tested working memory, executive functions and several sub-processes of procedural learning in all three studies. To control for possible confounding effects, we included age, gender and chronotype as covariates in our analyses. To test the amount of evidence either for associations or no associations between subjective sleep quality and cognitive performance, we calculated Bayes Factors that offer a way of evaluating the evidence against or in favor of the null hypothesis, respectively.

## Methods

### Participants

Participants were selected from a large pool of undergraduate students from Eötvös Loránd University. The selection procedure was based on the completion of an online questionnaire assessing mental and physical health status. Respondents reporting current or prior chronic somatic, psychiatric or neurological disorders, or the regular consumption of drugs other than contraceptives were excluded. In addition, individuals reporting the occurrence of any kind of extreme life event (e.g., accident) during the last three months that might have had an impact on their mood or daily rhythms were also excluded from the study.

The data was obtained from three different studies, each with a slightly different focus. Importantly, the analyses presented in the current paper are completely novel, none of the separate studies focused on the relationship between subjective sleep quality and cognitive performance. Forty-seven participants took part in Study 1^27^, 103 participants took part in Study 2^28^, and 85 participants took part in Study 3^29^. The descriptive characteristics of participants in the three studies are listed in Table 1. All participants were white/Caucasian. All participants provided written informed consent and received course credits for taking part. The studies were approved by the Research Ethics Committee of Eötvös Loránd University (201410, 2016/209). The study was conducted in accordance with the Declaration of Helsinki.

**Table 1:** Descriptive characteristics of participants.

Study	N	Age*Mean (SD)*	Gender	Years in education*Mean (SD)*	MEQ score*Mean (SD)*
Study 1	47	21.38 (1.79)	10 M/37 F	14.36 (1.58)	34.96 (6.69)
Study 2	103	21.62 (2.00)	30 M/73 F	14.50 (1.74)	33.99 (6.31)
Study 3	85	20.99 (1.59)	23 M/62 F	14.28 (1.60)	33.61 (5.68)

*Note:* M = male, F = female, MEQ = Morningness-Eveningness Questionnaire.

### Procedure

We conducted three separate studies on the association of subjective sleep quality and procedural learning, working memory, and executive functions in healthy young adults. The sleep questionnaires included in the studies and the timing of the procedural learning task slightly differed. While we assessed subjective sleep quality by PSQI and AIS in all three studies, in Study 2, we included further measures of subjective sleep quality as well: (1) a sleep diary to assess day-to-day general sleep quality and (2) Groningen Sleep Quality Scale (GSQS) to assess prior night’s sleep quality. To control for the potential confounding effect of chronotype, we also administered the Morningness-Eveningness Questionnaire (MEQ)^30,31^, henceforth referred to as morningness score because a larger score on this questionnaire indicates greater morningness.

In all three studies, PSQI and AIS sleep quality questionnaires and the MEQ were administered online, while the GSQS in Study 2 and the tasks assessing cognitive performance in all studies were administered in a single session in the lab. Due to technical problems, the data of six participants on executive functions are missing. To ensure that participants do the tests in their preferred time of the day, the timing of the session was chosen by the participants themselves (between 7 am and 7 pm). The timing of the sessions was normally distributed in all three studies, with most participants performing the tasks during the daytime between 11 am and 3 pm. The sleep diary in Study 2 was filled by the participants for at least one week, and to a maximum of two weeks, prior to the cognitive assessment that was scheduled based on the participants’ availability.

### Questionnaires and tasks

All cognitive performance tasks and subjective sleep questionnaires are well-known and widely used in the field of psychology and neuroscience (for details about each task and questionnaire, see Supplementary methods).

#### Subjective sleep quality questionnaires

To capture the general sleep quality of the last month, we administered the Pittsburgh Sleep Quality Index (PSQI)^14,32^ and the Athens Insomnia Scale (AIS)^15,33^. Additionally, in Study 2, we administered a Sleep diary^34^ to assess the sleep quality of the last one-two weeks, and the Groningen Sleep Quality Scale (GSQS)^35,36^ to capture the sleep quality of the night prior testing.

#### Cognitive performance tasks

Working memory was measured by the Counting Span task^37–40^. Executive functions were assessed by the Wisconsin Card Sorting Test (WCST)^41–43^. The outcome measure of the WCST task was the number of perseverative errors, which shows the inability/difficulty to change the behavior despite feedback. Procedural learning was measured by the explicit version of the Alternating Serial Reaction Time (ASRT) task (Figure S1, see also^44^). There are several learning indices that can be acquired from this task. Higher-order sequence learning refers to the acquisition of the sequence order of the stimuli. Statistical learning refers to the acquisition of frequency information embedded in the task. However, previous ASRT studies often assessed Triplet learning, which is a mixed measure of acquiring frequency and sequential information (for details, see Supplementary methods). In addition to these learning indices, we measured the average reaction times (RTs) and accuracy (ACC), which reflect the average general performance of the participants across the task, and the changes in RT and ACC from the beginning to the end of the task, which indicate general skill learning that occurs due to more efficient visuomotor and motor-motor coordination as the task progresses^45^.

### Data analysis

Statistical analyses were conducted in R 3.6.1^46^ using the lme4 package^47^. Bootstrapped confidence intervals and p-values were calculated using the boot package^48,49^. The data and analysis code can be found on the following link: https://github.com/nthun/performance_sleep_quality/

#### Analysis of the relationship between subjective sleep quality and cognitive performance

Subjective sleep quality scales (PSQI and AIS) were combined into a single metric, using principal component analysis. Then separate linear mixed-effect models were created for each outcome measure (i.e., performance metric), where the aggregated sleep quality metric (hereinafter referred to as sleep disturbance) was used as a predictor, and ‘Study’ (1, 2 or 3) was added as a random intercept. This way we could estimate an aggregated effect while accounting for the potential differences across studies. To control for possible confounding effects, we included age, gender and morningness score as covariates in our analyses. Thus, the estimates reported in the Results section are controlled for these factors.

As the residuals did not show normal distribution, we used bootstrapped estimates and confidence intervals, using 1000 bootstrap samples, from which we calculated the p-values^48,49^. Bayes Factors (BF_01_) were calculated by using the exponential of the Bayesian Information Criterion (BIC) of the fitted models minus the BIC of the null models – that contained the confounders only, and a random intercept by study^50^. The BF is a statistical technique that helps conclude whether the collected data favors the null-hypothesis (*H*0) or the alternative hypothesis (*H*1); thus, the BF could be considered as a weight of evidence provided by the data^51^. It is an effective mathematical approach to show if there is no association between two measures. In Bayesian correlation analyses, *H*0 is the lack of associations between the two measures, and *H*1 states that association exists between the two measures. Here we report BF_01_ values. According to Wagenmakers *et al*.^51^, BF_01_ values between 1 and 3 indicate anecdotal evidence for *H*0, while values between 3 and 10 indicate substantial evidence for *H*0. Conversely, while values between 1/3 and 1 indicate anecdotal evidence for *H*1, values between 1/10 and 1/3 indicate substantial evidence for *H*1. If the BF is below 1/10, 1/30, or 1/100, it indicates strong, very strong, or extreme evidence for *H*1, respectively. Values around 1 do not support either *H*0 or *H*1. Thus, Bayes Factor is a valuable tool to provide evidence for no associations between constructs as opposed to frequentists analyses, where no such evidence can be obtained based on non-significant results.

To test the association between the additional subjective sleep quality measures and cognitive performance in Study 2, we used robust linear regression, this time without random effects. We included the same potential confounders (age, gender, morningness score), and Bayes factors were calculated in the previously described way.

#### Analysis of the ASRT data

Performance in the ASRT task was analyzed by repeated-measures analyses of variance (ANOVA) in each study (for details of these analyses, see Supplementary methods). Based on these ANOVAs, Triplet learning, Higher-order sequence learning, and Statistical learning occurred in all three studies, both in ACC and RT (all *p*s < 0.001; for details, see Supplementary results and Figure S2).

## Results

### Cognitive performance in the three studies

The working memory capacity (measured by the counting span) and executive functions (measured by the number of perseverative errors in the WCST task) of the participants were in the standard range for their age^52,53^. The mean counting span for the entire sample was 3.59 (*SD* = 0.85) in the three studies. This average score represents a mid-range cognitive performance, as obtainable scores range from 1 to 6. The mean number of perseverative errors was 14.76 (*SD* = 5.27) in the three studies (no maximum score can be defined in this case). For procedural learning, mean scores were 26.48 (*SD* = 26.37) for RT Triplet learning, 16.63 (*SD* = 40.34) for RT Higher-order sequence learning, 16.74 (*SD* = 9.94) for RT Statistical learning, 359.88 (*SD* = 40.94) for average RT, and 31.13 (*SD* = 30.15) for RT general skill learning. Accuracy scores were as follows: 0.04 (*SD* = 0.03) for ACC Triplet learning, 0.02 (*SD* = 0.03) for ACC Higher-order sequence learning, 0.03 (SD = 0.03) for ACC Statistical learning, 0.90 (*SD* = 0.10) for average ACC, −0.02 (*SD* = 0.09) for ACC general skill learning, in all three studies. Note that for accuracy, these values represent proportions (e.g., the average ACC was 90%, hence 0.90), and the learning scores are difference scores (e.g., the ACC Triplet learning score shows that participants were on average 4% more accurate on high-frequency triplets compared to the low-frequency ones). All presented RT and ACC scores represent typical values in ASRT studies with healthy young adults.

We also provide descriptive data for Study 2 separately, as additional analyses were run on cognitive performance from this dataset and GSQS and sleep diary scores. In Study 2, the mean counting span was 3.65 (*SD* = 1.01), and the mean number of perseverative errors was 14.46 (*SD* = 6.37). For procedural learning in Study 2, mean scores were 33.04 (*SD* = 27.96) for RT Triplet learning, 28.53 (*SD* = 51.44) for RT Higher-order sequence learning, 18.77 (*SD* = 9.78) for RT Statistical learning, 348.29 (*SD* = 42.26) for average RT, and 39.30 (*SD* = 34.74) for RT general skill learning. Accuracy scores were as follows: 0.03 (*SD* = 0.02) for ACC Triplet learning, 0.01 (*SD* = 0.02) for ACC Higher-order sequence learning, 0.02 (*SD* = 0.02) for ACC Statistical learning, 0.94 (*SD* = 0.03) for average ACC, 0.02 (*SD* = 0.03) for ACC general skill learning.

Overall, these values represent a mid-range cognitive performance with a sufficient level of variability in the sample to conduct the planned analyses.

### Subjective sleep questionnaire scores in the three studies

The obtainable scores, means, standard deviations, and proportions of good, moderate and poor sleepers for each questionnaire are presented in Table 2. The mean scores of PSQI in the current sample were higher than the score of 1.91 for the same components in Buysse *et al*.^14^, and in the range or even higher than the global PSQI score (which aggregates seven components; *M* = 2.67) for the control participants, whose age was between 24 and 83 years. In the same study^14^, the participants with sleep disorders had a mean score of 4.78 for the three components of PSQI, suggesting that ~18% of the current sample had a score higher than the average score of sleep-disordered patients. The mean scores of AIS were somewhat higher than the mean score of 3 reported for a representative Hungarian adult sample in Novak *et al*.^33^. According to the cut-off score of 10 suggested in that paper, ~5% of our sample would fall into the diagnostic category of insomnia. However, according to a stricter cut-off score of 6 suggested by Soldatos, Dikeos & Paparrigopoulos^54^, up to 23% of the participants would have complaints comparable to those of insomniac patients. The mean of the GSQS score was lower than the mean score reported for a Hungarian sample of young adults (*M* = 4.70, *SD* = 1.78) in Simor *et al*.^35^. The mean of the Sleep diary score in Study 2 was comparable to the mean PSQI score of 1.3 for the same components for the control participants and lower than the score of 6.36 for the participants with sleep disorders in Buysse *et al*.^14^.

**Table 2:** Descriptive statistics of the subjective sleep questionnaire scores.

	Obtainable scores	Mean (SD)	Good sleepers	Moderate sleepers	Poor sleepers
			Scores (percentage of participants)		
PSQI	0–9				
All participants		2.99 (1.57)	0–1 (15.3%)	2–4 (66.4%)	5–8 (18.3%)
Study 2		2.54 (1.29)			
AIS	0–24				
All participants		3.98 (2.66)	0–2 (35%)	3–6 (50%)	7–17 (15%)
Study 2		3.41 (2.09)			
GSQS	0–14				
Study2		2.86 (2.87)	0–1 (40%)	2–7 (53%)	8–13 (7%)
Sleep dairy	0–12				
Study 2		1.38 (1.22)	0–1 (60%)	2–5 (40%)	

*Note:* PSQI = Pittsburgh Sleep Quality Index, AIS = Athens Insomnia Scale, GSQS = Groningen Sleep Quality Scale.

Although with some differences across questionnaires, these sleep scores suggest a moderate to poor sleep quality of the current sample, with about 15% of participants experiencing very poor sleep quality, comparable to those of patients with sleep disorders. Overall, all sleep measures used in the current study appear to have a sufficient level of variability to conduct the planned analyses.

### Combining sleep quality metrics

Principal component analysis was used to combine PSQI and AIS into a single ‘sleep disturbance’ metric. The Bartlett’s test of sphericity indicated that the correlation between the scales was adequately large for a PCA, χ^2^(235) = 84.88, *p* < 0.0001. One principal factor with an eigenvalue of 1.55 was extracted to represent sleep disturbance. The component explained 83.7% of the variance, and it was named ‘sleep disturbance’ as higher values of this metric show more disturbed sleep. The aggregated sleep disturbance index across the three studies ranged from -1.9 to 3.86.

### Associations between subjective sleep quality and cognitive performance

**Figure 1:**
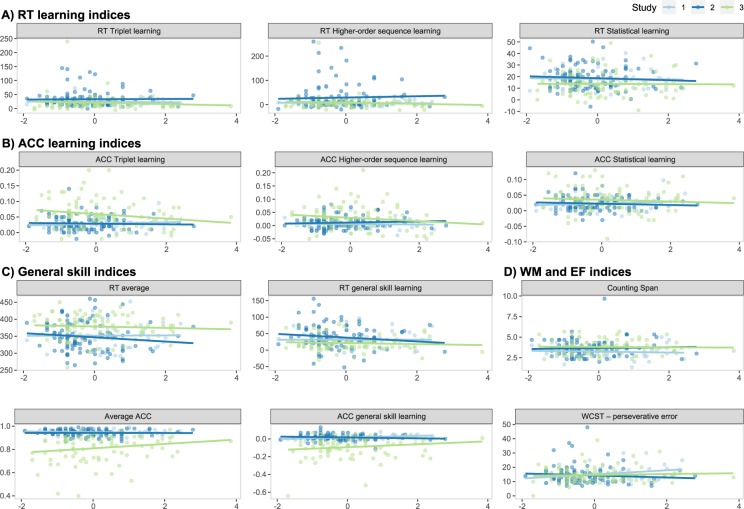
Association between sleep disturbance and cognitive performance metrics by study. Horizontal axes represent the sleep disturbance index, while vertical axes represent the outcome variables, with their names shown in the panel titles. The scatterplots and the linear regression trendlines show no association between subjective sleep quality and procedural learning indices in terms of reaction time (RT, **A**), or accuracy (ACC, **B**), general skill indices in terms of RT or ACC (**C**), and working memory and executive function indices (**D**).

As described above, to study the associations between subjective sleep quality and cognitive performance, separate linear mixed-effect models were created for each outcome measure (i.e., cognitive performance metric), where sleep disturbance was used as a fixed predictor, and ‘Study’ was added as a random intercept. Sleep disturbance did not show an association with any of the cognitive performance metrics (see Table 3 and Fig. 1). Bayes Factors ranged from 5.01 to 14.35, indicating substantial evidence for no association between subjective sleep quality and the measured cognitive processes^51^.

**Table 3:** The association of sleep disturbance with cognitive performance metrics.

Outcome	*β*	95% CI	df	*p*	BF_01_
**ACC learning indices**					
ACC Higher-order sequence learning	−0.041	[−0.18, 0.11]	205	0.58	12.28
ACC Statistical learning	−0.038	[−0.17, 0.09]	205	0.56	12.42
ACC Triplet learning	−0.067	[−0.19, 0.06]	205	0.30	8.50
**RT learning indices**					
RT Higher−order sequence learning	0.014	[−0.15, 0.16]	205	0.85	14.29
RT Statistical learning	−0.062	[−0.21, 0.07]	205	0.39	10.48
RT Triplet learning	−0.028	[−0.17, 0.12]	205	0.71	13.60
**General skill indices**					
ACC general skill learning	0.037	[−0.06, 0.13]	205	0.45	11.06
Average ACC	0.065	[−0.04, 0.17]	205	0.23	6.79
RT average	−0.019	[−0.17, 0.12]	205	0.80	14.05
RT general skill learning	−0.075	[−0.23, 0.07]	205	0.33	8.83
**WM and EF indices**					
Counting Span	−0.013	[−0.17, 0.14]	205	0.87	14.35
WCST – perseverative error	0.107	[−0.03, 0.24]	199	0.13	5.01

*Note*: The table shows standardized regression coefficients for sleep disturbance, where the ‘Study’ random intercept was included in separate linear mixed-effect models for each cognitive performance metrics. Age, gender, and morningness score were added as covariates. BF_01_ was derived from BIC (see the ‘Data analysis’ section for details). ACC = accuracy. RT = reaction time. WM = working memory. EF = executive function. WCST = Wisconsin Card Sorting Test.

To test whether AIS or PSQI scores separately are associated with cognitive performance, we performed similar analyses as for the sleep disturbance metric. Additionally, we also tested whether cognitive performance differed between “good” and “poor” sleepers as defined by the extremes in the overall PSQI score. For this analysis, we considered those with a score of 0 or 1 as good sleepers (N = 36), while those with a score of 5 to 8 as poor sleepers (N = 43), corresponding to approximately the upper and lower 15% of the data (see Table 2). These additional analyses (reported in the Supplementary results) are consistent with the above findings for the sleep disturbance metric, suggesting no relationship between subjective sleep quality and cognitive performance using these measures.

**Figure 2:**
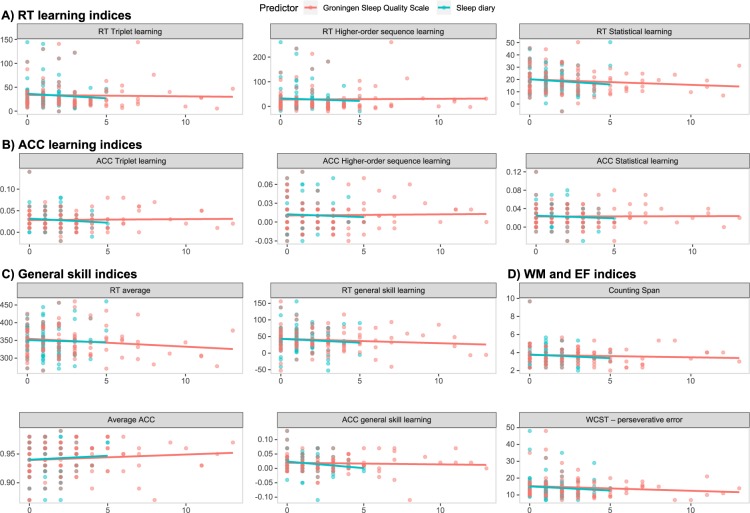
Association between sleep diary and GSQS scores and cognitive performance metrics. Horizontal axes represent the sleep disturbance index, while vertical axes represent the outcome variables, with their names shown in the panel titles. The scatterplots and the linear regression trendlines show no association between subjective sleep quality (measured with a sleep diary (blue) or the GSQS (red)) and procedural learning indices in terms of reaction time (RT, **A**), or accuracy (ACC, **B**), general skill indices in terms of RT or ACC (**C**), and working memory and executive function indices (**D**).

In Study 2, to investigate the associations between further subjective sleep quality questionnaires and cognitive performance, we created a separate linear mixed-effect model for each outcome measure (i.e., cognitive performance metric), and each additional sleep questionnaire (i.e., sleep diary and GSQS). Sleep diary scores did not show association with any of the cognitive performance metrics (all *p*s > 0.05, see Table 4 and Fig. 2). Bayes Factors ranged from 2.51 to 12.58, indicating, in all but one cases, substantial evidence for no association between subjective sleep quality and measures of cognitive performance^51^. The lowest value of 2.51 for ACC general skill learning also pointed to the same direction, indicating slightly weaker evidence for no association with subjective sleep quality.

**Table 4:** The association of sleep diary with cognitive performance metrics in Study 2.

Outcome	*β*	95% CI	*t*	df	*p*	BF_01_
**ACC learning indices**						
ACC Higher-order sequence learning	−0.077	[−0.28, 0.13]	−0.749	97	0.46	7.73
ACC Statistical learning	−0.031	[−0.24, 0.17]	−0.296	97	0.77	8.09
ACC Triplet learning	−0.111	[−0.31, 0.09]	−1.092	97	0.28	4.46
**RT learning indices**						
RT Higher-order sequence learning	−0.001	[−0.11, 0.11]	−0.025	97	0.98	9.76
RT Statistical learning	−0.205	[−0.41, 0.00]	−1.955	97	0.05	8.96
RT Triplet learning	−0.059	[−0.19, 0.07]	−0.917	97	0.36	11.28
**General skill indices**						
ACC general skill learning	−0.171	[−0.35, 0.01]	−1.866	97	0.07	2.51
Average ACC	0.035	[−0.18, 0.25]	0.317	97	0.75	8.94
RT average	−0.086	[−0.31, 0.13]	−0.764	97	0.45	12.79
RT general skill learning	−0.064	[−0.26, 0.14]	−0.623	97	0.53	7.10
**WM and EF indices**						
Counting Span	−0.065	[−0.26, 0.13]	−0.664	97	0.50	5.63
WCST – perseverative error	0.005	[−0.13, 0.14]	0.072	96	0.94	9.71

*Note*: The table shows standardized regression coefficients for sleep diary scores in separate linear mixed-effect models for each cognitive performance metrics. Age, gender, and morningness score were added as covariates. BF_01_ was derived from BIC (see ‘Data analysis’ section for details). ACC = accuracy. RT = reaction time. WM = working memory. EF = executive function. WCST = Wisconsin Card Sorting Test.

Similarly, GSQS scores did not show association with any of the cognitive performance metrics (all *p*s > 0.11, see Table 5 and Fig. 2). Bayes Factors ranged from 3.46 to 16.46, indicating substantial evidence for no association between subjective sleep quality and the measured cognitive processes^51^.

**Table 5:** The association of GSQS with cognitive performance metrics in Study 2.

Outcome	*β*	95% CI	*t*	df	*p*	BF_01_
**ACC learning indices**						
ACC Higher-order sequence learning	0.029	[−0.17, 0.23]	0.278	102	0.78	10.87
ACC Statistical learning	−0.001	[−0.20, 0.20]	−0.013	102	0.99	10.08
ACC Triplet learning	0.000	[−0.20, 0.20]	0.000	102	1.00	10.15
**RT learning indices**						
RT Higher-order sequence learning	−0.004	[−0.11, 0.10]	−0.070	102	0.94	10.14
RT Statistical learning	−0.105	[−0.32, 0.11]	−0.973	102	0.33	5.39
RT Triplet learning	−0.054	[−0.17, 0.07]	−0.866	102	0.39	16.46
**General skill indices**						
ACC general skill learning	0.040	[−0.13, 0.21]	0.452	102	0.65	12.28
Average ACC	0.156	[−0.05, 0.36]	1.466	102	0.15	5.16
RT average	−0.176	[−0.39, 0.04]	−1.617	102	0.11	3.46
RT general skill learning	−0.104	[−0.30, 0.09]	−1.039	102	0.30	5.85
**WM and EF indices**						
Counting Span	−0.062	[−0.26, 0.13]	−0.632	102	0.53	6.07
WCST – perseverative error	−0.009	[−0.13, 0.14]	−0.133	101	0.89	9.22

*Note*: The table shows standardized regression coefficients for GSQS scores in separate linear mixed-effect models for each cognitive performance metrics. Age, gender, and morningness score were added as covariates. BF_01_ was derived from BIC (see the ‘Data analysis’ section for details). ACC = accuracy. RT = reaction time. WM = working memory. EF = executive function. WCST = Wisconsin Card Sorting Test.

## Discussion

Our aim was to investigate the relationship between subjective sleep quality and cognitive performance in healthy young adults. Cognitive performance was tested in the domains of working memory, executive functions, and procedural learning. To provide more reliable results, we pooled data from three different studies, controlled for possible confounders, such as age, gender, and chronotype, and performed robust frequentists as well as Bayesian statistical analyses. We did not find associations between subjective sleep quality and cognitive performance measures using the robust frequentist statistical analyses. Moreover, the Bayes factors provided substantial evidence for no association between subjective sleep quality and measures of working memory, executive functions, and procedural learning. This pattern held when subjective sleep quality was reported retrospectively for a longer period (i.e., a month; with PSQI and AIS), as well as when monitored daily (for one to two weeks; with the sleep diary) or reported for the night prior to testing (with GSQS). These results suggest that neither moderately persistent nor transient subjective sleep quality is associated with cognitive performance in healthy young adults.

There are several factors to consider why subjective sleep quality showed no associations with cognitive performance in our sample of healthy young adults. First, it is possible that methodological issues contributed to the null effects. For example, having a lower range of obtainable scores on the selected subjective sleep quality and cognitive performance measures can limit the possibility of finding a relationship between these measures. Importantly, all measures that we used in the current study have been well-established in previous research and have a reasonable range of obtainable values. Although the sample choice of healthy young adults has naturally limited the range of scores on the used measures, our analyses showed a sufficient level of variability in all measures. Therefore, the obtained null results seem unlikely to be explained by such methodological issues.

Second, as we studied healthy university students, there may be a ceiling effect in subjective sleep quality. Sleep disturbance can be more prevalent in elderly populations and clinical disorders^14,33^. Consequently, variance and extremities in subjective sleep quality could be greater in these populations, while it can remain relatively low in healthy young adults. Nevertheless, previous research has found that university students are also prone to sleep disturbances, and in particular to chronic sleep deprivation^55^. Although with some variation across sleep questionnaires, most participants’ subjective sleep quality ranged from moderate to poor in our sample, with about 15% of participants experiencing very poor sleep quality similar to those of patients with sleep disorders. Thus, it seems unlikely that the obtained results are due to a ceiling effect in subjective sleep quality.

Third, it is possible that because young adults typically show a peak cognitive performance, poor subjective sleep quality may not have a substantial impact on it. In line with this explanation, the studies that reported associations between subjective sleep quality and cognitive performance^4,5,17^ focused primarily on adolescents, older adults, or clinical populations, where cognitive performance has not yet peaked or have declined. Further supporting this explanation, Saksvik *et al*.^56^ found in their meta-analysis that young adults are not as prone to the negative consequences of shift work as the elderly. Moreover, Gao *et al*.^57^ in a recent study showed that above-average cognitive abilities buffer against insufficient sleep durations. However, not all cognitive functions peak in adulthood: while previous studies have reported the best performance in working memory and executive functions in young adulthood^58–61^, some aspects of procedural learning (as measured by the ASRT task) has been shown to peak in childhood and to decline already around adolescents^44,62,63^. Consequently, a cognitive peak may explain finding no relationship between subjective sleep quality and aspects of working memory and executive functions, while this explanation for the measures of procedural learning seems unlikely.

Fourth, the conditions under which the data collection took place could have also contributed to the null results. We conducted our experiments during the term-time when the workload in the university is typically moderate. Moreover, students could choose the time of day for cognitive testing, and they may have chosen a time when they typically felt well-rested. There is evidence that performing in a preferred circadian time period can attenuate the effect of sleep disturbances^64^. Consistently, previous studies showed that participants exhibit better performance on working memory and executive functions tasks in their preferred time of day^65,66^. However, a recent study found that participants, in fact, exhibit weaker performance in procedural learning in their preferred time of day, and better performance in their non-preferred time of day, suggesting variability in the relationship between circadian effects and cognitive functions^67^. Additionally, independent of the time of day, participants may have perceived the session with the cognitive tasks as a testing situation and may have been motivated to show their best performance, compensating for any possible effect of poor subjective sleep quality. Indeed, there is evidence that highly motivated participants are less prone to the effect of sleep deprivation^68^. Thus, the time of testing and participants’ motivation may have contributed to our findings by potentially compensating for any negative effects of poor subjective sleep quality on cognitive performance.

Fifth, the relationship between sleep and cognitive performance can vary depending on what parameters of sleep are assessed. Associations between objective sleep quality (measured by actigraphy or electroencephalography) and various aspects of working memory, executive functions, and procedural learning have been frequently reported in previous studies (for a review, see^8,9^). Here we showed that subjective sleep quality is not associated with these cognitive functions, at least under the circumstances described above. As outlined in the Introduction, this dissociation suggests that objective and subjective sleep quality, although measure the same domains, do not necessarily capture the same aspects of sleep quality and sleep disturbances^11^. Subjective sleep quality may be estimated based on a combination of objective sleep parameters. Moreover, some objective parameters of sleep that contribute to cognitive performance may not be captured with self-reported instruments. For example, it is often reported that spindle activity or time spent in slow-wave sleep (SWS) or in rapid eye movement (REM) sleep is essential for memory consolidation^69–71^. Also, in laboratory sleep examinations, sleep quality is usually carefully controlled for several days prior to the examination. Potentially, the objective sleep parameters showing associations with cognitive performance may only be measured in these carefully controlled conditions (i.e., when sleep quality on the night of testing as well as in the preceding days are good). Hence, it is possible that while results with objective sleep quality may show how healthy sleep is related to cognitive functioning, results with subjective sleep quality may reflect how aspects of sleep disturbances are related to cognitive functioning.

Sixth, and relatedly, there could be differences in the association with cognitive performance within self-reported measures of sleep as well. In our study, we captured the perceived disturbances in initiating and maintaining sleep rather than the self-reported duration of sleep. While we found no associations between these measures of subjective sleep quality and cognitive performance, there is solid evidence that self-reported extreme sleep durations (both long and short sleep times) are associated with worse cognitive performance^18–20^. These findings suggest a dissociation between sleep quality as measured by extreme self-reported sleep durations and other types of sleep quality disturbances.

Seventh, it is possible that while interindividual differences in subjective sleep quality do not contribute to at least some aspects of cognitive performance, intraindividual fluctuations do. The possible importance of intraindividual rather than interindividual differences was also suggested by Ackerman *et al*.^72^ in a large study, in which contrary to previous studies they showed no associations between declarative memory consolidation and objective sleep parameters. Further studies are warranted to test whether day-to-day variations in subjective sleep quality predict day-to-day changes in cognitive performance.

Finally, our paper has some limitations. As mentioned above, it is possible that investigating populations more susceptible to sleep disturbances or cognitive performance problems could yield different results and the lack of associations could be specific to healthy young adults. Furthermore, it would be interesting to test whether individual differences in other factors (for example, interoceptive ability, i.e., how accurately one perceives their own body sensations) influence the relationship between subjective sleep quality and cognitive performance.

## Conclusions

In conclusion, we showed that self-reported, subjective sleep quality is not associated with working memory, executive functions, and various aspects of procedural learning in a relatively large sample of healthy young adults. These findings were supported not only by frequentist statistical analyses but also by Bayes factors that provided substantial evidence for no associations between these functions. Importantly, however, our findings do not imply that sleep per se has no relationship with these cognitive functions; instead, it emphasizes the dissociation between subjective and objective sleep quality. We believe that our approach of systematically testing the relationship between self-reported sleep questionnaires and a relatively wide range of cognitive functions can inspire future systematic studies on the relationship between subjective/objective sleep parameters and cognition. Within healthy young adults, future studies are warranted to probe the relationship between subjective sleep quality and cognitive performance assessed in the non-preferred time of day, include other aspects of cognitive functions, and test intraindividual, day-to-day variations in the relationship between sleep and cognitive performance.

## Supplementary information

Supplementary information .

## Data Availability

The dataset and analysis code of the current study are available in the Open Science Framework repository, https://osf.io/hcnsx/.
